# Dielectric properties, thermal properties, blood perfusion rate: which biophysical parameter is the leading source of variability of microwave ablation models in lung?

**DOI:** 10.1080/02656736.2026.2685124

**Published:** 2026-06-15

**Authors:** Anna Bottiglieri, Punit Prakash

**Affiliations:** Department of Biomedical Engineering, The George Washington University, Washington, DC, USA

**Keywords:** Microwave ablation, lung cancer, bioheat transfer modeling, tissue biophysical properties, tumor-tissue blood perfusion

## Abstract

**Background:**

The large variability in outcomes following microwave ablation (MWA) of pulmonary tumors highlights a need for quantitative methods to assess 3D temperature profiles accounting for tissue-specific biophysical characteristics. The goal of this study is to identify the least and most influential biophysical parameters on the ablation zone.

**Methods:**

A computational bioheat transfer model was built to simulate a 2.45 GHz microwave applicator positioned within a 2 cm diameter tumor. Simulations were conducted using 60 W - 10 min energy settings and considering low and high values of: 1) relative permittivity, 28.3–50.4 and effective conductivity, 1.0–1.9 (Sm^−1^); 2) volumetric heat capacity, 1.3–3.5 (MJm^−3^K^−1^) and thermal conductivity 0.2–0.4 (Wm^−1^K^−1^); 3) blood perfusion rate of tumor periphery-parenchyma, 5–9 (kgm^−3^s^−1^). Length (L_60_) and diameter (D_60_) of the 60 °C isotherm, volumetric thermal coverage of the tumor without (TM0) and with 5 mm margin (TM5) were quantified.

**Results:**

The variabilities in the volumetric heat capacity and blood perfusion rate at the tumor-lung parenchyma boundary yield differences between 5.7 – 11.4% (TM5), 3.1–4.6 mm (D_60_), 3.0–4.4 mm (L_60_). Variabilities in baseline values of dielectric properties yield 3% (TM5) and 1.2 mm (D_60_ and L_60_) differences.

**Conclusion:**

Thermal properties and blood perfusion between tumor and surrounding tissue are the most impactful parameters on achieving adequate treatment margin following lung MWA. This quantitative analysis provides insight on the tissue biophysical properties that warrant deeper experimental assessment in order to inform treatment planning of lung MWA.

## 1. Introduction

Image-guided microwave ablation (MWA) is an available treatment modality along with stereotactic body radiation therapy (SBRT) for primary and metastatic lung tumors, typically of a diameter less than 3 cm [1], when patients are not candidates for surgical resection [2–5]. A clinical MWA procedure involves the positioning of a microwave (MW) applicator (13–17 gauge needle, 2.45 GHz or 915 MHz operating frequency) within the tumor under imaging guidance. Electromagnetic energy is radiated from the tip of the MW applicator within the tissue for a time between 5 and 10 min at a power level ranging between 40 and 60 W depending on the tumor size and relative distance from surrounding anatomical structures, such as pleura, heart, major blood vessels and airways [6].

In lung, ablation zones are generally assessed via computed tomography (CT) imaging (with or without contrast). Immediately after the procedure, the tissue adjacent to where the MW applicator was positioned is surrounded by a layer of higher attenuation where the underlying parenchyma is still visible, i.e., ground-glass opacity (GGO). Histological analysis showed that the inner layer of the treated tissue correlates with loss of tissue architecture and alveolar effusion, whilst the surrounding layer presents with mixed effects including alveolar congestion, hemorrhage and viable cells [7]. An MWA procedure in lung is considered completed when the imaging-assessed GGO encompasses the tumor and a minimum circumferential margin of 5 mm of non-tumor tissue [8,9]. However, there exists a general consensus that CT or MR imaging scans acquired earlier than 1-month post-ablation are unlikely to provide definitive assessment of the ablated volume [10]. Indeed, in the weeks after the ablation procedure, processes such as fibrosis and cavitation change the GGO appearance and the early-assessed ablation zone gradually decreases.

The variabilities in the anatomical and biophysical characteristics across tumors together with limitations in assessing ablation margin immediately after the procedure are all factors contributing to the large variability of the ablation procedure outcome. Retrospective studies reported variabilities in the ablated volume ranging between ~ 3–14 cm^3^ [11] across 72 patients (113 ablations) when using the same energy delivery settings; and deviations from the ablation volumes predicted by the device manufacturer ranging between −95 to −5 cm^3^ [12]. This large variability poses challenges for achieving treatment outcome, limiting unnecessary thermal dose to lung parenchyma and the risk of deleterious effects to surrounding vital organs [13].

In current practice, treatment planning relies on device manufacturer-based estimates of ablation zone extent, which are typically derived from experiments in *ex vivo* animal tissue and thus do not account for case-specific considerations, including perfusion-mediated cooling effects. Physics-based computational models are currently under investigation for potential use in treatment planning of ablation procedures, including for MWA procedures in lung. The motivation of studies exploring physics-based computational models of MWA is to advance treatment planning approaches based on manufacturer estimates, by incorporating more patient-specific information [14–18].

**Figure 1.**
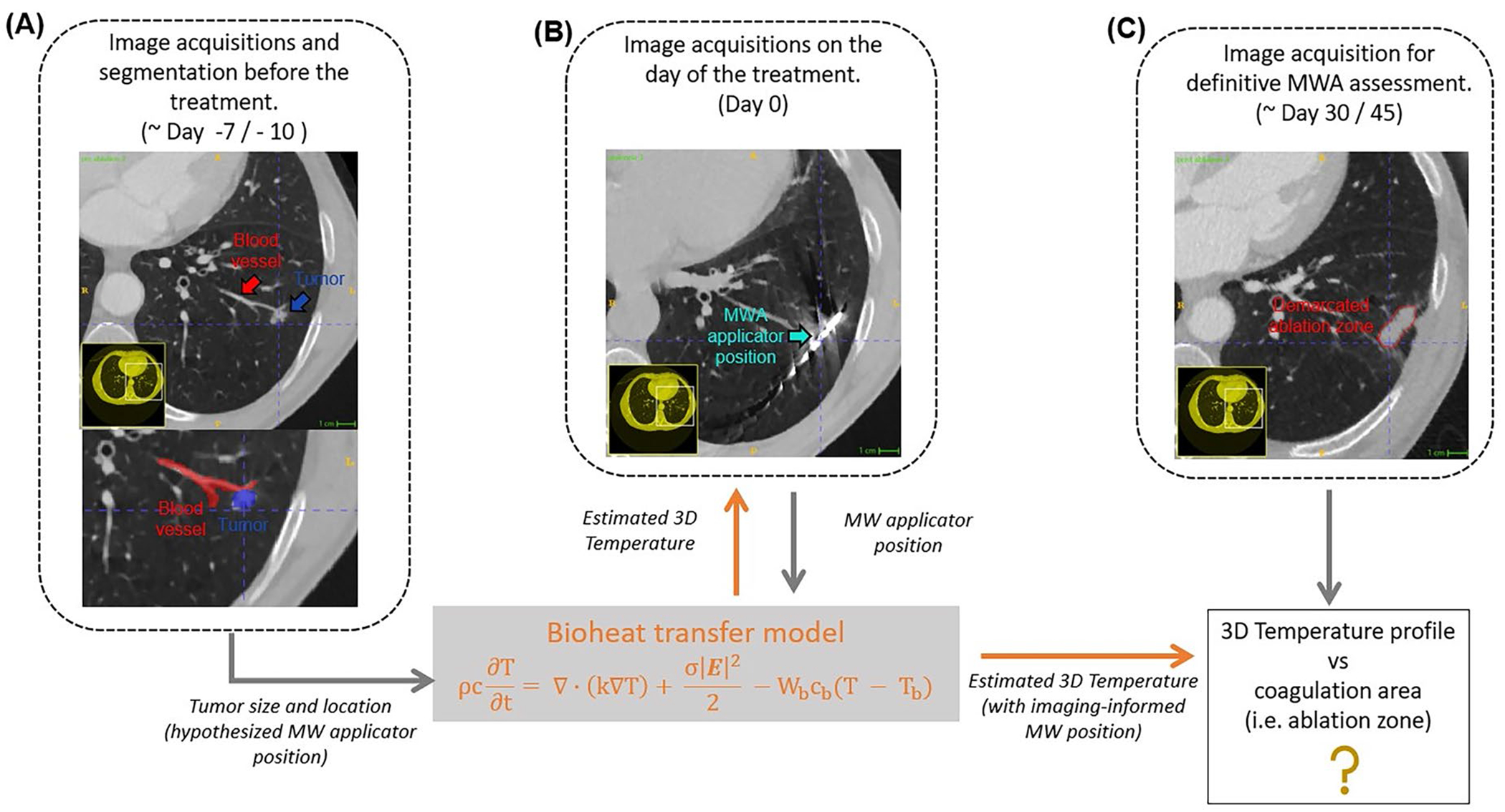
Imaging-informed modeling framework describing how a bioheat transfer model can be implemented within a clinical workflow for planning MWA treatments of lung tumors. (A) Pre-treatment imaging and segmentation of the tumor and nearby anatomical structures. (B) Bioheat transfer computational model including anatomical parameters of the tumor and information of MW applicator design and position. (C) Comparison between the computed 3D temperature profile and ablation zone estimated on the CT imaging typically acquired between 30 and 45 days after thermal ablation. (The CT images are part of the data from a previous study [14] conducted in our group).

In a recent retrospective clinical study [14], an imaging-informed computational framework was adopted to inform bioheat transfer simulations. Pretreatment CT imaging information of size and shape of each specific lung tumor and position of the MW applicator were included. It was found that within a cohort of 50 patients, the computed ablation volume overlaps the CT imaging measures with a statistically significant higher coefficient of similarity (0.55 ± 0.14) as compared with the manufacturer geometric model (0.46 ± 0.19). Figure 1 shows an example of how an imaging-informed computational modeling approach could be incorporated within the clinical workflow, similar to [14] and employed for treatment planning of MWA in lung tumors.

The findings shown in [14] encourage further studies on the capabilities of physics-based computational models of MWA in lung to provide reliable estimates of the 3D temperature profile on a tumor-specific basis. In clinical settings, computed estimates of the ablation zone based on anatomical, biophysical and physiological characteristics of each tumor have the potential to reduce the inter-patient variability of the ablation outcome and/or support clinical decisions on subsequent treatment approaches.

Methods to assess biophysical and physiological properties of lung tumors and adjacent parenchyma on patient-specific basis are not clearly streamlined. Consequently, most studies employ baseline values (i.e., at physiologic temperature) of dielectric, thermal and blood perfusion properties of the tumor and lung parenchyma independent of the possible inter-patent variabilities [14,19,20].

Between 2023 and 2024, the first dielectric properties measurements in resected human lung tissues at room temperature (baseline values) and at 2.45 GHz became available [21,22]. Those studies show that depending on the condition of the tissue (e.g., normal lung parenchyma, cancerous tissues, fibrotic tissue), relative permittivity and effective conductivity at 2.45 GHz – operating frequency of most of the commercially available clinical MWA systems – vary between 28.3–50.4 and 1.0–1.9 (Sm^−1^), respectively. These variabilities are the largest assessed so far in the baseline values of the tissue dielectric properties [22]. Recent experimental studies [23,24] showed that thermal conductivity and volumetric heat capacity measured in lung and liver at room temperature (baseline values) vary between 0.2–0.5 (Wm^−1^K^−1^) and 1.3–3.5 (MJm^−3^ K^−1^), respectively. In addition [23,24], showed that thermal conductivity and volumetric heat capacity also vary substantially as a function of the temperature. Finally, qualitative and quantitative assessment of CT perfusion imaging of lung and lung tumors showed variability in the averaged values of blood perfusion rate between 39.1–111.6 (ml min^−1^100g^−1^) depending on the tumor type and location within the lung (e.g., upper and lower lobe) [25–28].

It is expected that temperature profile and the growth of the ablation are affected by the variabilities in the biophysical properties of the tissues involved during the heating [29,30]. Therefore, information limited to the size and shape of the tumor, energy delivery settings and position of the MWA applicator alone, might not be sufficient for accurate assessments of the ablation zone. For clinical use of modeling, incorporating patient-specific estimates of tissue properties may afford more refined treatment planning and assessment of lung MWA procedures.

The goal of the analysis conducted in this study is to identify among the biophysical parameters of the bioheat transfer models (Figure 1), those yielding highest variabilities on the estimates of the ablated tumor and non-tumor tissue volume in lung. We use the most recent published experimental data of thermal and dielectric properties and clinical imaging-derived information of blood perfusion rate with a focus on lung tissues, to quantify the influence of the variabilities in the baseline values of (1) relative permittivity and electrical conductivity, (2) volumetric heat capacity and thermal conductivity and (3) blood perfusion rate on the diameter and length of the ablation zone and volumetric thermal coverage of the tumor volume with and without 5 mm minimal margin. The results of this analysis are intended to provide a roadmap of the biophysical parameters that more than others drive the MWA outcome in lung, hence toward which the focus of forthcoming experimental studies might be most informative.

## 2. Methodology

### 2.1. Geometry

**Figure 2.**
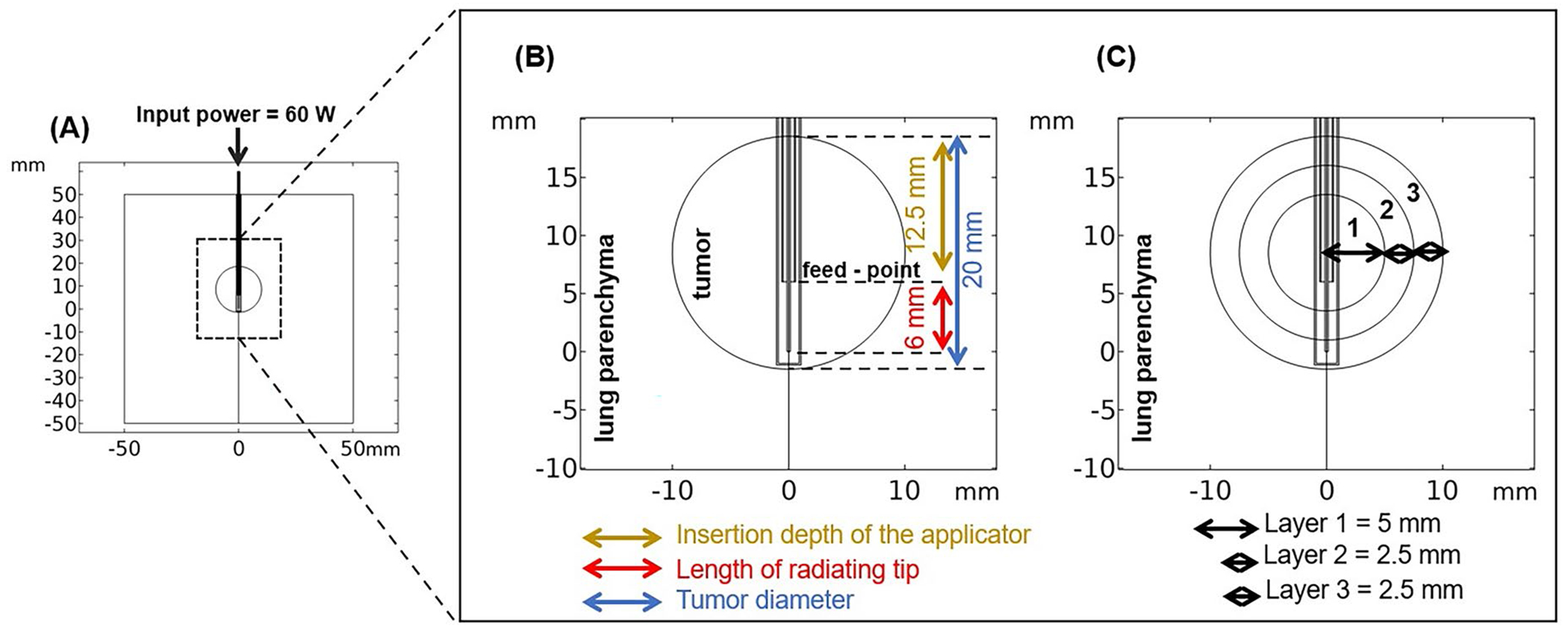
(A) Symmetrical geometry including a 20 mm diameter tumor enclosed within the lung parenchyma domain (*r* = 50 mm, *z* = 100 mm) and a microwave applicator with a 6 mm length radiating element and an insertion depth of approximately 2/3 (12.5 mm) the diameter of the tumor domain; (B) same symmetrical geometry and MW applicator position where different layers within the tumor have been created for different levels of blood perfusion rate increasing from Layer 1 (*r* = 5 mm) to layer 3 (*r* = 2.5 mm as well as for layer 2).

Figure 2(A) shows a geometry resembling a spherically-shaped tumor target, surrounding lung parenchyma and an MW applicator with z-axis of symmetry. The water-cooled MW applicator incorporating a coaxial monopole antenna (coaxial cable: outer conductor diameter = 1.2 mm, dielectric diameter = 0.9 mm, inner conductor diameter = 0.3 mm; external catheter diameter = 2.1 mm) with a 6 mm long radiating tip was centrally placed within a 20 mm diameter tumor as illustrated in Figure 2(B). The simplified 20 mm diameter spherical geometry was selected as a reference scenario in order to focus the study on the sensitivity of the ablation zone to the variabilities in tissue biophysical and physiological properties.

The overall dimensions of the geometry, 100 mm height and 100 mm width, were chosen to minimize the effects of the electromagnetic traveling wave reflected at the boundary of the geometry. A convergence test was conducted to identify the optimal size of the mesh elements similar to the approach shown in [31]. The final minimum and maximum mesh dimensions are 0.01 and 2.2 mm.

Convective heat transfer coefficient *h* = 500 (Wm^−2^K^−1^) and reference temperature *T* = 10 °C were assigned to the external wall of the MW applicator and the tissue geometry [32,33] to model the cooling effect of the circulating water. Scattering electrical boundary conditions were applied on all external surfaces of the geometry. The initial temperature for all domains of the geometry is 37 °C. Microwave input power was assigned to the distal end of the MW coaxial cable geometry (Figure 2(A)).

The homogenous tumor domain (Figure 2(B)) was modified to a three-layers structure (Figure 2(C)) and different values of blood perfusion rate were assigned to each layer (see 2.2. Methodology section for more details on the rationale for the specific values).

### 2.2. Biophysical parameters and metrics

Coupled electromagnetic-thermal simulations solved Helmholtz’s equation (Equation (1)) that calculates the distribution of the time-harmonic electric field (***E***) at 2.45 GHz and the dissipated electromagnetic power (*Q_emw_*) within the tissue.


(1) $$\nabla^{2}E+\beta_{0}^{2}\left(\varepsilon_{r}\left(T\right)-\frac{j\sigma \left(T\right)}{\omega \varepsilon_{0}}\right)E=0\;Q_{emw}=\frac{1}{2}\sigma \left(T\right){\left|E\right|}^{2}$$

In Equation (1), **E** is the electric field, β_0_ is the wavenumber in free space (m^−1^), ε_r_(T) and σ (T) are temperature-dependent relative permittivity and effective conductivity (Sm^−1^) of the tissue; ω = 2π*f* is the angular frequency (rads^−1^), where *f* in this study is 2.45 GHz, and ε_0_ is the permittivity in free space. The term Q_emw_ represents the electromagnetic heat source in the coupled bioheat transfer equation (Equation (2)).


(2) $$\rho c\left(T\right)\frac{\partial T}{\partial t}=\nabla \cdot k\left(T\right)\nabla T+Q_{emw}\left(T\right)-c_{bl}W_{bl}\left(T\right)\cdot \left(T-T_{bl}\right)$$

In Equation (2), ∇⋅k(T)∇T and $C_{bl}W_{bl}\left(T\right)\cdot \left(T-T_{bl}\right)$ are the terms describing the thermal diffusion of the heat and perfusion mediated tissue cooling, respectively; ρc is the volumetric heat capacity (Jm^−3^K^−1^), *k* is the thermal conductivity (Wm^−1^K^−1^), c_bl_ is the specific heat capacity of blood (Jkg^−1^K^−1^), W_bl_ is the blood perfusion rate (kgm^−3^s^−1^) and T_bl_ is the temperature of the blood (37 °C).

All simulations were conducted using an applied input power of 60 W for 10 min, which is in the range of energy delivery settings typically used in clinical MWA procedures [12,14,34].

This study focuses on the sensitivity of MW ablation in lung to the variabilities of the baseline values of each biophysical parameter in Equation (1) and Equation (2). Specifically, the analysis accounts for:

1. Low and high baseline values of relative permittivity and effective conductivity, ε_r_ = 28.3; σ = 1.00 (Sm^−1^) and ε_r_ = 50.4; σ = 1.9 (Sm^−1^). These values are the lowest and highest values of relative permittivity and effective conductivity among all combined measurements of dielectric properties on human lung tissue following surgical resection reported in [21,22]. For this first part of the analysis, we assumed baseline values for blood perfusion rate = 5 (kgm^−3^s^−1^), volumetric heat capacity = 3.5·10^6^ (Jm^−3^K^−1^) and thermal conductivity = 0.4 (Wm^−1^K^−1^).
2. Low and high values of volumetric heat capacity, ρc = 1.3·10^6^ and ρc = 3.5·10^6^ (Jm^−3^K^−1^), and thermal conductivity, k = 0.2 and k = 0.4 (Wm^−1^K^−1^). These values were taken from the measured values in bovine liver [24] and porcine lung [23]. We decided to include values related to liver as well, given the paucity of measurements of tissue thermal properties and given that the large temperature range (T > 95 °C) relevant to assess changes in thermal properties is available only for the study conducted in liver tissue. In addition, CT imaging features of diagnosed tumors show variability in solid vs sub-solid components of the tumor, many of which present a predominantly solid component [35]. Thus, it is reasonable to conceive that for a range of lung tumors, thermal properties are similar to those of liver and other solid tissues. For this second part of the sensitivity analysis, we assumed blood perfusion = 5 (kgm^−3^s^−1^), relative permittivity = 50.4, effective conductivity = 1.9 (Sm^−1^).
3. Values of the blood perfusion rate are based on the published studies conducted in clinical settings [25–28]; in those studies blood perfusion rate of lung tissues was assessed using perfusion CT imaging. We selected the studies reporting a similar protocol of imaging acquisition: 30 s breath hold, 25–30 s duration of the imaging scan, 1 s acquisition time interval. The blood perfusion rate assigned to the homogenous model (Figure 2(B)) was chosen based on the calculated average value among all data reported in the selected studies. In particular, 5 (kgm^−3^s^−1^) value is used to account for the blood perfusion up to 56 °C, in order to assess the independent effect of variabilities of dielectric properties and thermal properties on the ablation zone (see Sections 3.1 and 3.2). The 56 < T < 61 °C temperature interval over which blood perfusion rate is reduced from the baseline value to 0 kg m^−3^ s^−1^ to model blood perfusion cessation is similar to previous studies [36,37]. However, tumor blood perfusion is spatially heterogenous. The heterogenous model (Figure 2(C)) is a representation of the layer-by-layer assessment method applied to CT-imaging acquisitions of lung tumors reported in [38]. The CT-imaging informed method presented in [38], showed that the lowest HU voxels indicating low blood perfusion are located in the center of the tumors and that HU tends to increase toward the periphery of the tumor. Similarly, in the ‘heterogenous model’ lowest and highest blood perfusion rates were assigned to layer 1 and layer 3 respectively (Figure 2(C)), representing the gradual increase of perfusion from the core to the periphery of the tumor. To assess the impact of variability of blood perfusion at tissue-tumor boundary, in one case layer 3 and parenchyma were assumed to have equal perfusion, 5 (kgm^−3^s^−1^); in the second case a higher perfusion was assigned to the parenchyma domain, 9 (kgm^−3^s^−1^), as compared to the value 5 (kgm^−3^s^−1^) assigned to layer 3.

Table 1 provides range of variabilities of the baseline values and temperature-dependent trends for dielectric properties [39,40], thermal properties [24] and blood perfusion rate [41].

For each variable listed in Table 1, MWA outcome was quantified using the following metrics:

- length (L_60_) and diameter (D_60_) of the T = 60 °C isotherm at the end of simulated MWA (10 minutes). The 60 °C threshold is a surrogate indicator for coagulative necrosis occurring in tissue [42,43] during heating, and as such L_60_ and D_60_ are the metrics that are typically used to evaluate the extent of the ablation zone [44,45].
- percentage volumetric thermal coverage of the tumor with 0 mm (TM0) and 5 mm margin (TM5), similar to the metrics used in [14,34] to assess clinical outcome of MWA in lung.

A visual description of the metrics is provided in the (Supplement Material Figure S1). For the qualitative analysis of the temperature maps, we considered the 60 °C isotherm to estimate the coagulated tissue volume and the 45 °C isotherm to visualize the profile of the tumor and non-tumor tissue at risk of non-lethal thermal effects [36].

## 3. Results

**Figure 3.**
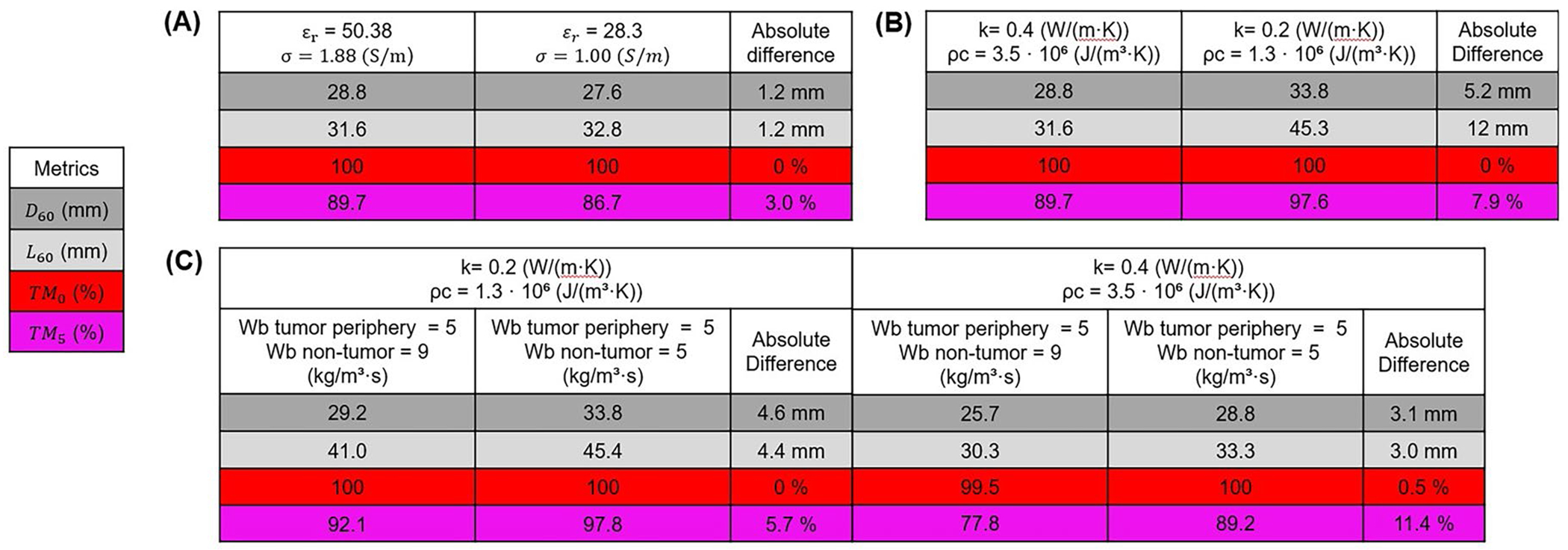
Values of diameter, D_60_, and length, L_60_, of the ablation zone demarcated by the 60 °C isotherms and the volumetric metrics TM0 and TM5 assessed at 10 min. MWA (input power = 60 W) in the case of (A) high vs low dielectric properties; (B) high vs low thermal properties; (C) Blood perfusion parenchyma > Blood perfusion tumor periphery (layer 3) for low thermal properties; (D) Blood perfusion parenchyma > Blood perfusion tumor periphery (layer 3) for high thermal properties.

Figure 3 shows the quantitative assessment of the sensitivity of D_60_ and L_60_, TM0 and TM5 metrics to variabilities of baseline values of dielectric properties (A) and thermal properties (B) and differences in blood perfusion (C). The absolute differences between each considered scenario are also provided.

### 3.1. What is the influence of dielectric properties?

Figure 3(A) shows that the difference (Δ) in the baseline values of relative permittivity (Δ = 22.1) and effective conductivity (Δ = 0.9 Sm^−1^) yielded a variability of 1.2 mm in both length (L_60_) and diameter (D_60_) and 3% in the TM5. In both scenarios of dielectric properties, the thermal coverage of 2 cm tumor (TM0 = 100%) was reached, whilst the TM5 remains below < 90% indicating that a 5 mm treatment margin is not ubiquitously reached under the selected energy settings, 60 W for 10 min. Overall, the quantitative assessment of computed thermal profiles reveals that the sensitivity of the ablation profile to the variabilities in the baseline values of the dielectric properties is negligible. Similarities in the thermal profile resulting from simulations considering low and high dielectric properties scenarios can also be qualitatively assessed from the temperature maps provided in the (Supplement Figure S2). Additional simulations for *low* and *high* dielectric properties scenarios were conducted using an MW applicator geometrical model that includes a 21 mm choke similar to the design illustrated in [46] and using different power and time settings (50 W for 5 min). Under these new settings, the impact of the baseline values of the dielectric properties was less than 2.5 mm in D_60_ and less than 2 mm in L_60_ (Figure S3, Table S1). Thus, the variabilities in the baseline values of the dielectric properties on the diameter of the ablation zone needed to achieve adequate margin was again found to be negligible when considering a different antenna design and applied energy settings.

### 3.2. What is the influence of thermal properties?

Figure 3(B)shows that the difference (Δ) in the baseline values of thermal conductivity and volumetric heat capacity is Δ = 0.2 (Wm^−1^K^−1^) and Δ = 2.2 · 10^6^ (Jm^−3^K^−1^), respectively. The differences in the baseline values of the thermal properties yielded 5.2 mm and 12 mm variabilities in D_60_ and L_60_, respectively. No variation was observed in the TM0, which is 100% for both cases. However, the difference in the TM5 metric between low and high thermal properties was 7.9%, which is approximately 2.6 times higher than the absolute difference in TM5 observed in the case of variability in the dielectric properties baseline values (Figure 3(A)).

**Figure 4.**
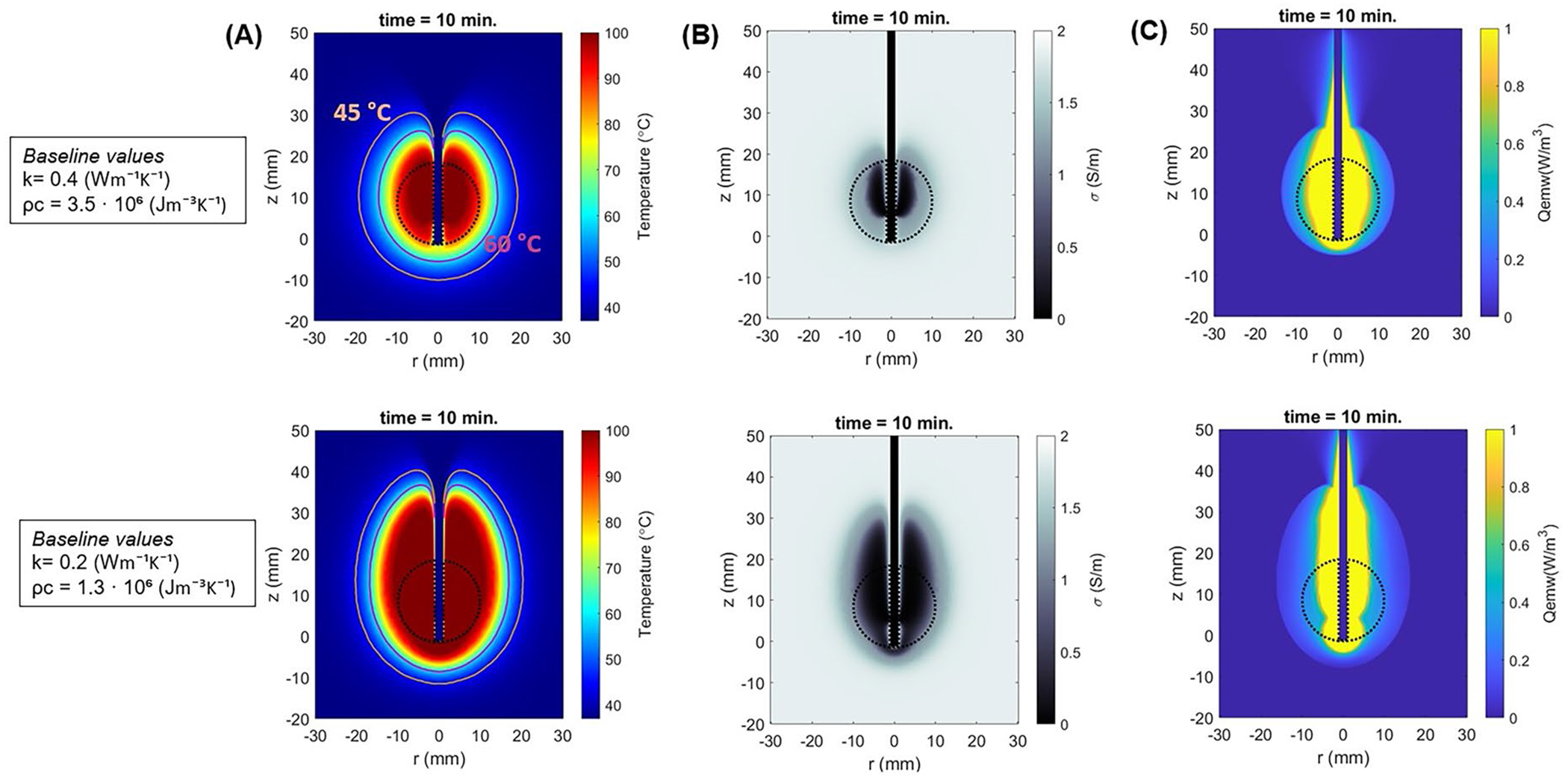
Comparison of the distribution of (A) temperature; (B) tissue electrical conductivity and; (C) absorbed electromagnetic power at 10 min MWA (input power 60 W) between high (first row) and low (second line) thermal properties. Baseline values, ε_r_ = 50.4, σ = 1.9 (Sm^−1^) for the temperature-dependent dielectric properties model and homogenous blood perfusion rate, 5 (kgm^−3^ s^−1^), up to 56 °C were employed for this comparison.

In Figure 4, the profiles of the temperature (A), effective conductivity (B) and absorbed electromagnetic power (C) are compared between the two scenarios of high (first row) and low (second row) values of thermal properties. Figure 4(A) shows that at 10 min, the aspect ratio (AR = D_60_/L_60_) of the ablation zone estimated by the 60 °C isotherm is ~0.7 in the case of low thermal properties, resulting in a thermal profile 43% longer (L_60_) and only 17% larger (D_60_) as compared to the case of high thermal properties (AR ~ 0.9).

Figure 4(B) shows that the profile of the effective conductivity, which is a temperature-dependent biophysical parameter (Table 1), is visibly affected by the two different values (high vs low) of volumetric heat capacity on the rate of increase of the temperature (dT/dt). Indeed, the extent of tissue where the effective conductivity decreased to more than half of the initial value, from 1.9 (S/m) to below 0.8 (S/m), is limited to a region measuring ~9 mm in diameter and ~10 mm in length in the case of high volumetric heat capacity (slow rate of heating), in contrast with ~14 mm diameter and ~21 mm length of the tissue for low volumetric heat capacity (high rate of heating). Given the relationship between the dissipated electromagnetic power within the tissue and effective conductivity shown in Equation (1), the difference in the profiles of effective conductivity inevitably mirrors the difference in the profiles of the absorbed electromagnetic power between the two scenarios of thermal properties. In a scenario of low thermal properties, because of the rapid rate of heating and thus rapid decline of the effective conductivity of the tissue, the electromagnetic power is primarily dissipated along the length of the MW applicator rather than contributing to the radial growth of the ablation zone; the increase of the temperature in the radial dimension is further limited by the relatively low value of thermal conductivity. Changes in the complex permittivity of tissue during heating, which are more rapid in the case of low volumetric heat capacity, lead to changes in the power absorption profile in tissue over the course of the ablation [39]. Because the tissue impedance is inversely proportional to the complex permittivity, when the relative permittivity decreases the electric field propagates toward the path of high relative permittivity (low tissue impedance), which is along the longitudinal axis of the applicator (negative z-axis) rather than forward within the tissue (positive z-axis).

### 3.3. What is the influence of blood perfusion rate?

For both low and high thermal properties examined earlier, Figure 3(C) shows the quantitative assessment of the influence of the variability of the blood perfusion rate within the tumor as compared to the non-tumor surrounding tissue.

**Figure 5.**
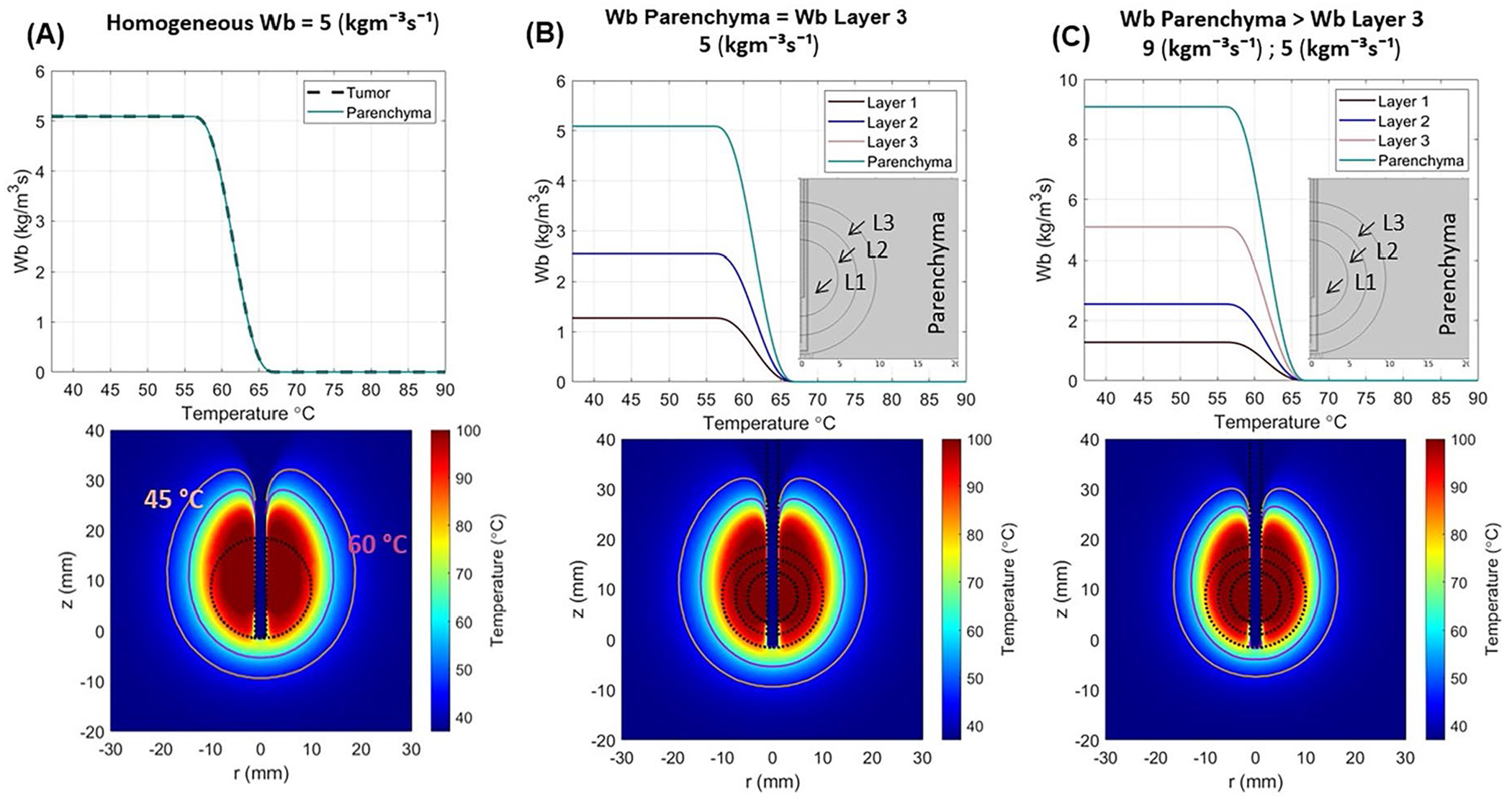
Temperature profile at 10 min of MWA (input power = 60 W) for three different scenarios of blood perfusion rate (Wb): (A) Wb = 5 (kgm^−3^s^−1^) in both the tumor and parenchyma domains; (B) model including three- layers structure (layer mode) with different values of blood perfusion within the tumor assuming no difference in Wb between tumor outer region (layer 3) and parenchyma (overlapping curves); (C) layer mode blood perfusion model within the tumor assuming Wb of the parenchyma > Wb of tumor outer region (layer 3). Baseline values of BHE dielectric and thermal parameters are ε_r_ = 50.4, σ = 1.9 (Sm^−1^), *k* = 0.4 (Wm^−1^K^−1^), ρc = 3.5 · 10^6^ (Jm^−3^K^−1^).

**Figure 6.**
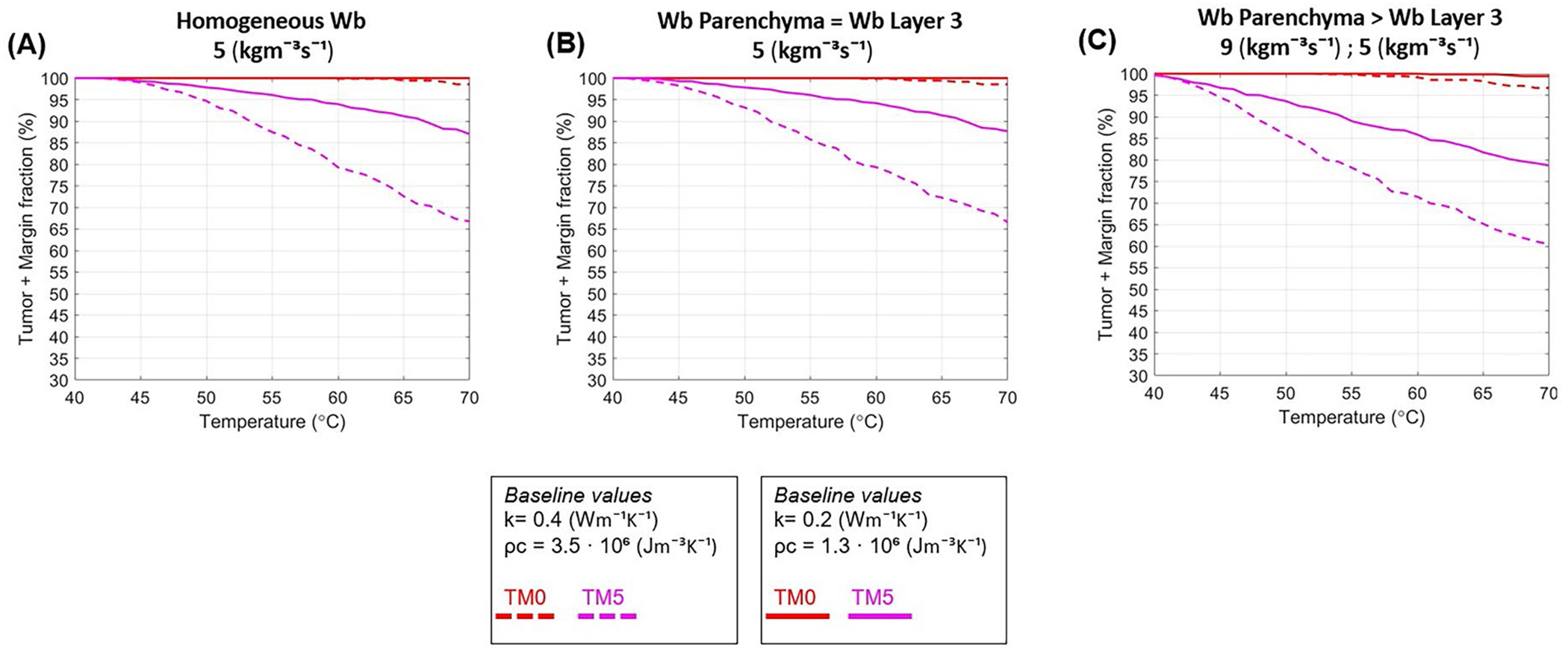
Graph of the volumetric thermal coverage of the tissue as a function of the temperature, namely temperature-volume histograms, illustrating variability in the thermal coverage of the tumor (TM0) and tumor + 5 mm minimum margin (TM5) in the cases of: (A) Wb = 5 (kgm^−3^s^−1^) in both the tumor and parenchyma domains; B) layer mode blood perfusion model within the tumor assuming Wb of the parenchyma = Wb of tumor outer region (layer 3); (C) layer mode blood perfusion model within the tumor assuming Wb of the parenchyma > Wb of tumor outer region (layer 3) and high (dotted lines) vs low (solid lines) thermal properties. Baseline values of BHE dielectric parameters are ε_r_ = 50.4, σ = 1.9 (Sm^−1^).

In the case of no difference in the blood perfusion rate between the periphery of the tumor (Layer 3) and the adjacent lung parenchyma (i.e., Wb tumor periphery = Wb non-tumor), L_60_, D_60_, TM0 and TM5 are not appreciably different from the values in Figure 3(B), for each corresponding scenario of thermal properties. In this scenario of contiguity of blood perfusion at the edge of the tumor, i.e., Wb tumor periphery = Wb non-tumor, Figures 5 and 6 show that the heterogeneities in blood perfusion values from the innermost layers of the tumor (Layer 1 and Layer 2) play a negligible role in the variability of the ablation zone and volumetric thermal coverage as compared to the homogenous perfusion scenario. This result can be explained by the evidence that the central (layer 1) and intermediate (layer 2) zones of the tumor are largely above 56 °C – the temperature threshold at which most of the vasculature can be considered coagulated. Thus, the perfusion-mediated cooling effect on the ablation zone boundary is mainly driven by the contrast in the blood perfusion rate between the periphery of the tumor (layer 3) and the adjacent lung parenchyma.

When the blood perfusion rate of the surrounding parenchyma is higher (9 kgm^−3^s^−1^) than the value at the periphery (layer 3) of the tumor (5 kgm^−3^s^−1^), the ablation zone decreases from 28.8–33.8 mm range to 25.7–29.2 mm in diameter (D_60_) and from 33.3–45.4 mm range to 30.3–41.0 mm in length (L_60_), depending on the underlying thermal properties of the tissue. The implications of the reduced dimensions of the ablation zone are particularly relevant when the volumetric thermal coverage is assessed. Figure 5(C) shows a visibly smaller temperature profile at 10 min as compared to the temperature profiles obtained when blood perfusion rate at the periphery of the tumor equals the value at the adjacent non-tumor tissue (Figure 5(A,B)).

The TM0 ~ 100% indicates that ablation of the 2 cm diameter tumor can be virtually achieved using energy settings of 60 W power for 10 min, independent of the variabilities in the thermal properties and blood perfusion rate. In contrast, TM5 decreases by about 11.4% in the case high thermal properties and 5.7% in the case of low thermal properties (Figure 3(C)), when the blood perfusion of the surrounding parenchyma is higher than the value in the tumor.

Figure 6 shows the combined effect of the variabilities in the thermal properties and blood perfusion rate on the evolution of the volumetric thermal coverage, presented as temperature-volume histograms in analogy with the dose-volume histogram (DVH) widely used in medical physics discipline to assess the radiation dose delivered to the planned target volume. Figure 6 reveals differences in the trend of TM5 within 40–70 °C temperature range, depending on the variabilities in the thermal properties and blood perfusion rate. In the scenarios of homogenous blood perfusion rate at the boundary between parenchyma and tumor, Figure 6(A) and Figure 6(B) show that in the case of high thermal properties (dotted lines) 94% of the TM5 volume exceeds 50 °C, 80% exceeds 60 °C and a further decline to 66% at a threshold of 70 °C, as compared to 97% (50 °C), 95% (60 °C) and 88% (70 °C) in the case of low thermal properties (solid lines). The difference in the volumetric thermal coverage between Figure 6(A) and Figure 6(B) was less than 1% for all temperatures thresholds between 40–70 °C; this result confirms that blood perfusion heterogeneities in the innermost regions of the tumor (layer 1 and layer 2) play a negligible role in the evolution of the volumetric thermal coverage as a function of the temperature.

Figure 6(C) shows that when blood perfusion rate of the surrounding tissue is higher than within the tumor, TM5 decreases at faster rates compared to the previous scenarios (Figure 6(A,B)) reaching values of: 85% (50 °C), 72% (60 °C) and 60% (70 °C) in the case of high thermal properties and 94% (50 °C), 85% (60 °C) and 79% (70 °C) in the case of low thermal properties. It is worthwhile noting that the cooling effect of the regional blood perfusion at the boundary between the tumor and surrounding tissue, results in 15–30% of the volume encompassing the tumor and 5 mm margin (TM5) below 60 °C of which 6–15% remains below 50 °C and ~ 5% below 45 °C. If we were to interpret this result through a clinical lens, it would signify a relatively high risk of viable tumor cells and that an additional ablation, if feasible, might be considered.

## 4. Discussion and conclusions

The overarching question posed in this study is: what are the biophysical parameters (Figure 1) playing a major role on the final ablated tissue volume relative to the tumor volume? To answer this question, a physics-based computational approach was employed providing quantitative and qualitative assessment of the sensitivity of the MWA outcome to variabilities of 1) dielectric properties, 2) thermal properties and 3) blood perfusion rates. This study seeks to contextualize the results of the sensitivity analysis within the clinical goal of achieving an adequate ablation margin. To this end, volumetric thermal coverage of the tumor with (TM5) and without (TM0) 5 mm margin was evaluated along with length (L_60_) and diameter (D_60_) of the ablation zone. This study attempts to provide a roadmap that could be used to design forthcoming *ex vivo* and/or *in vivo* experimental studies for the characterization of lung tumor and non-tumor tissue biophysical parameters toward informing patient-specific treatment planning of lung MWA.

The results of the first part of the sensitivity analysis focused on the independent effect of dielectric properties that were previously measured in resected human lung tissues. This analysis revealed a maximum 1.2 mm difference (on the order of less than the size of two pixels on CT imaging) in both length and diameter of the ablation zone and maximum 3% difference in the volumetric thermal coverage volume without (TM0) and with 5 mm margin (TM5) (Figure 3(A)). Because of the temperature-dependent nature of the dielectric properties (Table 1), the large differences of relative permittivity, Δ = 22.1 and effective conductivity, Δ = 0.9 (Sm^−1^) observed at *T* ~ 37 °C, reduces to Δ = 12 and Δ = 0.6 (Sm^−1^) at *T* ~ 90 °C. During MWA, temperature in the tissue next to the MW applicator (~ 3 mm radial distance) rises to 90 - 95 °C generally in the first 60–90 s of heating. The resultant changes of the dielectric properties alter the power absorption profile, thereby considerably slowing the rate of radial growth of the ablation zone. A similar biophysical behavior of the tissue in response to the increase of the temperature has been noted in a previous study on liver tissue [39]. By the end of the ablation procedure the effect of the variabilities in the baseline values of the dielectric properties is largely exhausted and the ablation growth continues via diffusion of heat (first term, right side in Equation (2)) radially toward the periphery of the tumor. The findings of the first part of this study suggests that available experimental studies of dielectric spectroscopy in lung tissues [21,22] provide solid knowledge for initializing temperature-dependent functions of the dielectric properties for clinically relevant physics-based models of lung MWA. Similar conclusions can be derived using a different MW applicator design i.e., a fully-cooled monopole antenna equipped with a choke; from these additional simulations (Figure S3, Table S1) the variability in the dielectric properties at the baseline has an impact of maximum 2.2 mm diameter and 1.6 length on the final temperature profile and less than 1.5% in volumetric thermal coverage which has comparatively little influence on achieving treatment margins.

The second part of the sensitivity analysis focusing on the effect of variabilities in measured values of thermal properties on ablation zone, showed that volumetric heat capacity in concert with the thermal conductivity shape the final temperature profile. In particular, this analysis highlighted the significance of the variabilities of the volumetric heat capacity on the shaping of the temperature profile (Figure 4(A)). By affecting the rate of heating (left side Equation (2)), volumetric heat capacity of the tissue influences the rate at which the dielectric properties decrease with the increase of the temperature.

Taking together the results of the effects of dielectric and thermal parameters, this analysis showed that the distribution of the effective conductivity within the tissue (Figure 4(B)) and, as a consequence, the distribution of the absorbed electromagnetic energy (Figure 4(C)) is highly affected by the underlying volumetric heat capacity of the tissue, more than the inherent differences of the baseline values of the dielectric properties. In this study, the temperature-dependent characterization of thermal properties measured in *ex vivo* liver tissue were used to approximate high water content lung tumors, however this remains an assumption that requires further experimental studies. Indeed, given the prominent role played by the volumetric heat capacity and, to some extent by the thermal conductivity, further experimental studies characterizing the temperature-dependent profile of thermal properties of lung tissue are needed, particularly of resected human tissue samples.

The third and final part of this sensitivity analysis shows that spatial heterogeneities in the blood perfusion rate within the tumor and surrounding lung parenchyma yield variabilities between 3–5 mm in both diameter (D_60_) and length (L_60_) of the ablation zone and between 5–12% in the volumetric coverage of the tumor and 5 mm margin (TM5), depending on the baseline thermal properties (Figure 3(C)). The analysis also reveals that the blood perfusion in the central/intermediate regions of the tumor (layer 1 and layer 2 in Figure 2(C)) play a negligible or minimal role in the growth of the ablation zone for a 2 cm diameter tumor, under the settings of 60 W input power for 10 min. The three-layers model of blood perfusion used in this study, although simplified, attempts to model the variabilities in the fraction of solid component within lung tumors and the implications for the underlying blood perfusion [47]. Additionally, the case scenario of higher blood perfusion in lung parenchyma as compared to the periphery of the tumor (layer 3) is a representation to evaluate the impact of the physiology of the background tissue, particularly in proximity to the tumor, that might be different between primary and secondary tumors. Notably, for tumors larger than 2 cm and/or different energy delivery settings, the impact of the blood perfusion at the boundary between the tumor and lung parenchyma on the ablation zone might be different; this further supports the rationale for designing future studies to estimate blood perfusion rates, particularly at the boundaries of the tumor. Similarly, we used an energy-delivery protocol that is generally used in clinical procedures; specific results may vary for different energy settings and/or ablation devices.

The volumetric thermal dosimetry assessment (Figure 6) further highlights that the capability to achieve adequate circumferential margin during MWA in lung, or the risk of excessive heating, is influenced by the variabilities in the thermal properties of the tumor and in the blood perfusion rate at the edge between the tumor and non-tumor tissue. Furthermore, this study provided an example of how physics-based computational methods can enable a nuanced quantitative analysis of thermal ablation outcome by assessing not only the fraction of coagulated tissue volume but also the fraction of tissue volume vulnerable to sublethal thermal effects (Figure 6). This aspect might be particularly relevant for thermal ablation procedures in lung, where GGO appearance is the imaging feature commonly used as a surrogate for evaluating the completion of the treatment. However, reliability of GGO imaging defects to estimate the ablated tissue volume is variable across patients [35]. Given the unique challenges that lung tissues pose for the assessment of adequate ablation margin, there is a rationale for devoting future research effort to advance physics-based modeling by exploring opportunities that available CT imaging might present for systematic evaluation of the ablation zone [27,48].

In order to focus this study on the sensitivity of the volumetric thermal coverage to the variability of the parameters in the bioheat transfer equation (Equation (2)) we used a single tumor diameter, single MW applicator position and single energy delivery setting. However, the methods we illustrated and the metrics we adopted can be extended to studies using different combinations of tumor diameter, energy delivery parameters and device position.

In conclusion, this study shows that:

- Thermal properties and blood perfusion rate at the edge of the tumor are the primary biophysical parameters that influence the extent and the shape of the ablation zone in lung and the final volumetric thermal coverage.
- The variabilities in the baseline values of the dielectric properties have a negligible impact compared to thermal properties and blood perfusion on the estimated volumetric thermal coverage including adequate ablation margin.
- The assessment of the ablation zone based only on the length and diameter of the ablation zone might not be sufficient to estimate success of MWA procedures in lung. Volumetric thermal dosimetry studies enabled by employing computational methods could provide relevant information for MWA treatment planning.

## Supplementary Material

Supp 1

Supplemental data for this article can be accessed online at https://doi.org/10.1080/02656736.2026.2685124

## Figures and Tables

**Table 1.** Parameters of the bioheat transfer equation – volumetric heat capacity, thermal conductivity, electrical conductivity, relative permittivity, blood perfusion rate – corresponding baseline values and temperature-dependent trends.

BHE parameters	Symbol (unit)	Baseline values (range)	Ref.	Temperature-dependent trends
Volumetric heat capacity	ρc (Jm^−3^K^−1^)	1.3e - 6, 3.5e - 6	[23,24]	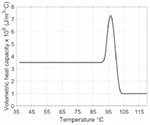
Thermal conductivity	k (Wm^−1^K^−1^)	0.2, 0.4	[23,24]	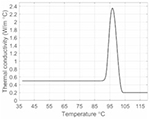
Electrical conductivity	σ (Sm^−1^)	1.0, 1.9	[21,22]	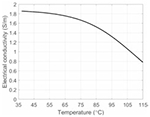
Relative Permittivity	ε_r_ (−)	28.3, 50.4	[21,22]	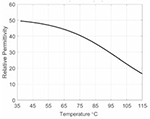
Blood perfusion rate	W_b_ (kgm^−3^s^−1^)	^a^1, 3, 5, 9	[25–28]	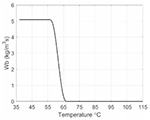

a Baseline blood perfusion rate was fixed to 5 (kgm^−3^s^−1^) for all cases assuming homogenous blood perfusion model (no layers). Values from 1 to 9 (kgm^−3^s^−1^) were considered only for the analysis of the influence of homogenous vs layers-mode blood perfusion rate (see Section 2.2 of the manuscript and Figure 5).

## Data Availability

The data that support the findings of this study are available from the corresponding author, [A. B.], upon request.
