# Supplementary material for: Dielectric properties, thermal properties, blood perfusion rate: which biophysical parameter is the leading source of variability of microwave ablation models in lung?
Source: Int J Hyperthermia. Author manuscript; Available in PMC 2026 Aug 2. (PMC13428987; doi:10.1080/02656736.2026.2685124)
Supplement: Supp 1 [file NIHMS2189866-supplement-Supp_1.docx]

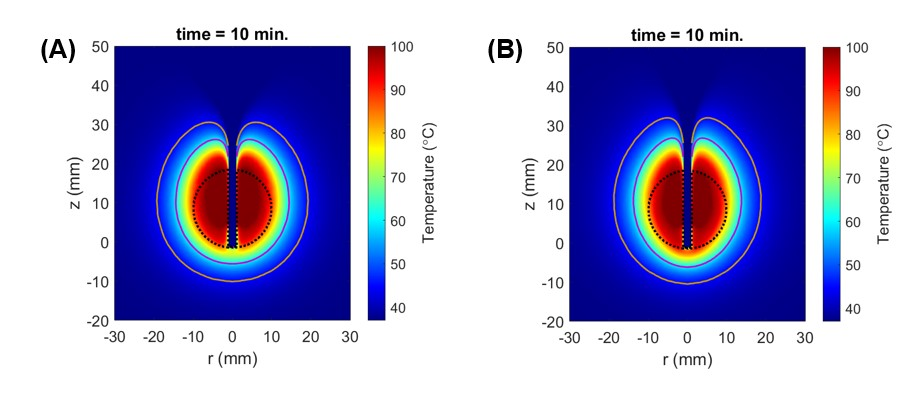


**Figure S2**. Qualitative comparison of the temperature profile at the end (10 min.) of MWA (input power 60 W) between (A) high: εᵣ = 50.38 σ = 1.88 (Sm¯¹) and (B) low: εᵣ = 28.3 σ = 1.0 (Sm¯¹) baseline values of dielectric properties.


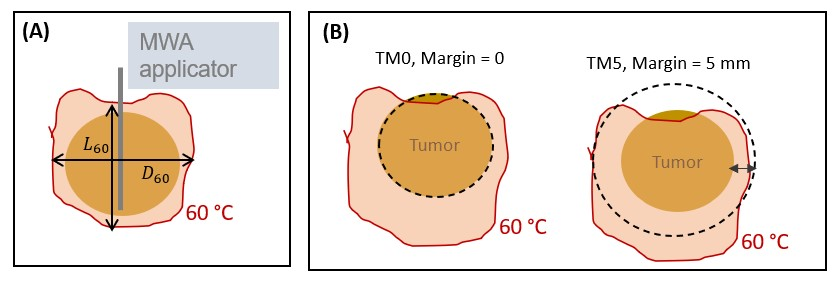


**Figure S1**. Representation of (A) Length and Diameter of the 60°C isotherm and (B) thermal coverage with delineated (dotted line) margin = 0 (TM0) and margin = 5 (TM5) adopted to quantify the effect of the variabilities in the biophysical parameters in the bioheat equation on the variabilities of the estimated outcome of microwave ablation in lung.

**Table S1**. Summary of compiled simulations for 20 mm tumor geometry using 60 W and 10 minutes energy parameters and a fully-cooled monopole MW applicator model.

| No. | Model type | Parameter description | Symbol | Baseline values |
| --- | --- | --- | --- | --- |
| 1 | Homogenous | Dielectric properties | *σ* (Sm^-1^)  *ε_r_* (-) | 1.0  28.3 |
|  |  |  |  |  |
|  |  |  |  |  |
|  |  | Thermal properties | *ρc* (Jm^-3^K^-1^)  *k* (Wm^-1^K^-1^) | 3.5e-6  0.4 |
|  |  | Blood perfusion | W_b_ (kgm^-3^s^-1^) | 5 |
| 2 | Homogenous | Dielectric properties | *σ* (Sm^-1^)  *ε_r_* (-) | 1.9  50.4 |
|  |  | Thermal properties | *ρc* (Jm^-3^K^-1^)  *k* (Wm^-1^K^-1^) | 3.5e-6  0.4 |
|  |  | Blood perfusion | W_b_ (kgm^-3^s^-1^) | 5 |
| 3 | Homogenous | Dielectric properties | *σ* (Sm^-1^)  *ε_r_* (-) | 1.9  50.4 |
|  |  | Thermal properties | *ρc* (Jm^-3^K^-1^)  *k* (Wm^-1^K^-1^) | 1.3e-6  0.2 |
|  |  | Blood perfusion | W_b_ (kgm^-3^s^-1^) | 5 |
| 4 | Heterogenous  (3 layers model) | Dielectric properties | *σ* (Sm^-1^)  *ε_r_* (-) | 1.9  50.4 |
|  |  | Thermal properties | *ρc* (Jm^-3^K^-1^)  *k* (Wm^-1^K^-1^) | 3.5e-6  0.4 |
|  |  | Blood perfusion | W_b_ (kgm^-3^s^-1^) | Layer 1 = 1  Layer 2 = 3  Layer 3 = 5  Parenchyma = 5 |
| 5 | Heterogenous  (3 layers model) | Dielectric properties | *σ* (Sm^-1^)  *ε_r_* (-) | 1.9  50.4 |
|  |  | Thermal properties | *ρc* (Jm^-3^K^-1^)  *k* (Wm^-1^K^-1^) | 1.3e-6  0.2 |
|  |  | Blood perfusion | W_b_ (kgm^-3^s^-1^) | Layer 1 = 1  Layer 2 = 3  Layer 3 = 5  Parenchyma = 5 |
| 6 | Heterogenous  (3 layers model) | Dielectric properties | *σ* (Sm^-1^)  *ε_r_* (-) | 1.9  50.4 |
|  |  | Thermal properties | *ρc* (Jm^-3^K^-1^)  *k* (Wm^-1^K^-1^) | 3.5e-6  0.4 |
|  |  | Blood perfusion | W_b_ (kgm^-3^s^-1^) | Layer 1 = 1  Layer 2 = 3  Layer 3 = 5  Parenchyma = 9 |
| 7 | Heterogenous  (3 layers model) | Dielectric properties | *σ* (Sm^-1^)  *ε_r_* (-) | 1.9  50.4 |
|  |  | Thermal properties | *ρc* (Jm^-3^K^-1^)  *k* (Wm^-1^K^-1^) | 1.3e-6  0.2 |
|  |  | Blood perfusion | W_b_ (kgm^-3^s^-1^) | Layer 1 = 1  Layer 2 = 3  Layer 3 = 5  Parenchyma = 9 |


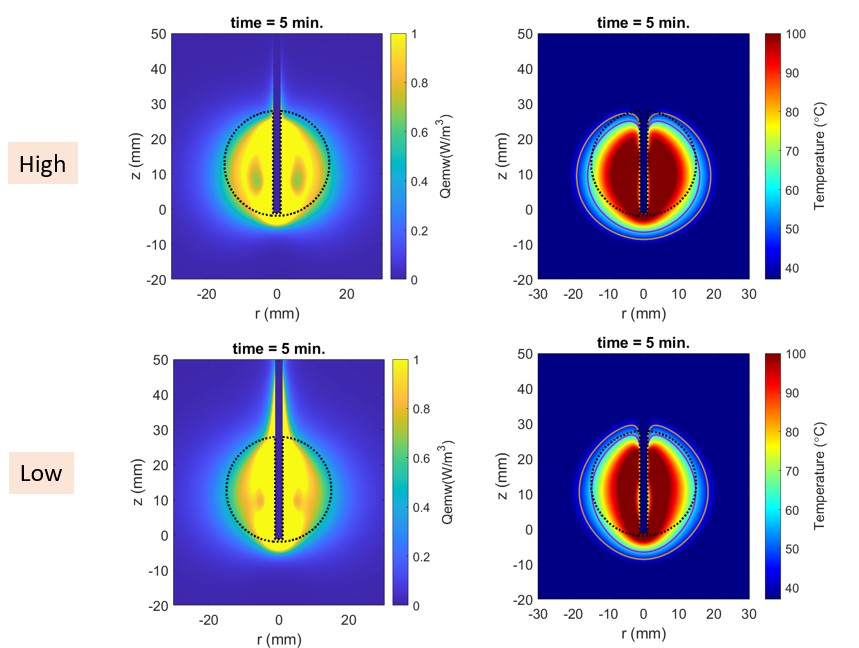


**Figure S3**. Qualitative comparison of the absorbed electromagnetic power (Q_emw_) and temperature profile at the end (5 min.) of MWA (input power 50 W) between (A) high: εᵣ = 43 σ = 1.7 (Sm¯¹) and (B) low: εᵣ = 21 σ = 0.8 (Sm¯¹) baseline values of dielectric properties. The MWA applicator geometry includes a water-cooled monopole and a 21 mm choke.

**Table S2.** Diameter and Length of the 60 °C isotherm at the end (5 min.) of MWA (input power 50 W) delivered with a MWA applicator design including a 21 mm choke assuming in one case high and in the other low dielectric properties yielding Δ = 22 and Δ = 0.9 (S/m) at the baseline conditions (T = 37 °C).

| **Time** | **5 min.** | | | |
| --- | --- | --- | --- | --- |
| **Dielectric**  **Properties** | D_60_ (mm) | L_60_ (mm) | TM0  (%) | TM5  (%) |
| *High*  ε ~ 43, σ ~ 1.7 (S/m) | 31.8 | 31.6 | 100 | 87.6 |
| *Low*  ε ~ 21, σ ~ 0.8 (S/m) | 29.6 | 33.2 | 100 | 88.7 |
| Diff  High - Low | 2.2 | -1.6 | 0 | 1.1% |
